# 472. A Scoping Review of Medical Education Innovations Presented at IDWeek

**DOI:** 10.1093/ofid/ofaf695.160

**Published:** 2026-01-11

**Authors:** Amy E Meyer, Darcy Wooten, Christian Hendrix, Joshua Nordman, Miguel A Chavez, Nathan Nolan, Nicolo Cabrera, Reid Goodman, Tri Pham, Gayathri Krishnan

**Affiliations:** Washington University in St. Louis, St. Louis, MO; Washington University in St. Louis, St. Louis, MO; BJH WashU, St Louis, MO; Washington University in St Louis, Saint Louis, MO; Washington University in St. Louis, St. Louis, MO; Washington U Sch of Med, St. Louis, Missouri; Washington University in St. Louis, St. Louis, MO; Washington University School of Medicine, St. Louis, Missouri; Washington University in St. Louis School of Medicine, St. Louis, MO; Washington University in St. Louis, St. Louis, MO

## Abstract

**Background:**

The Infectious Diseases (ID) Medical Education (MedEd) category at IDWeek is a relatively recent addition, and its scope and quality have not been systematically assessed. We conducted a scoping review of IDWeek MedEd abstracts (2020–2024) to characterize content areas, learner type, instructional strategies, and outcomes, to inform future ID MedEd research priorities.

**Figure 1:**
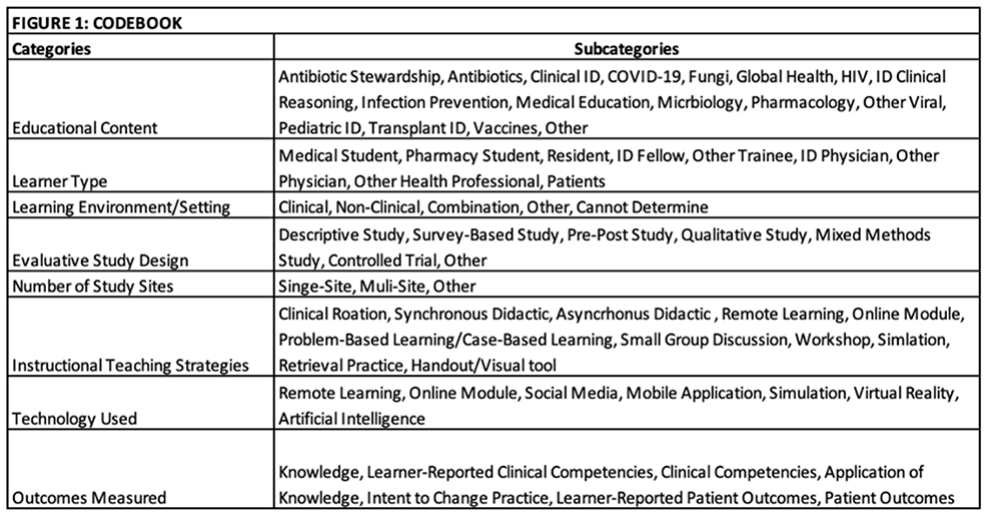
Codebook of categories

Figure 1 describes the categories in the Codebook used for data collection in this scoping review.

**Figure 2:**
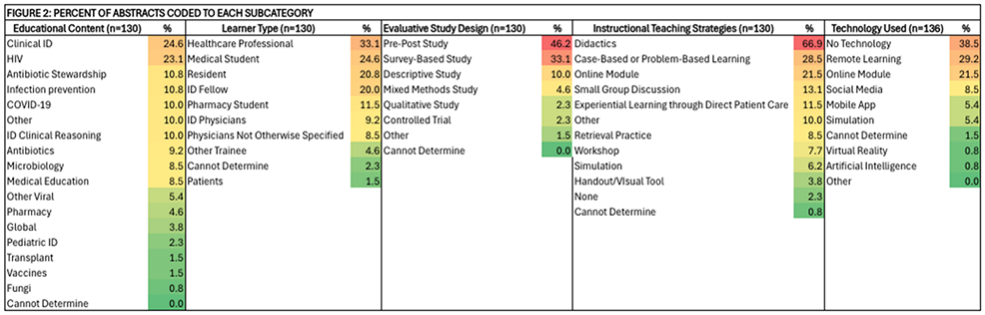
Percent of Abstracts Coded to Each Subcategory

Figure 2 is a heatmap that illustrates percentages of abstracts that were classified into each category using the Codebook.

**Methods:**

Guided by Constructivist learning theory, we used a deductive coding approach, expanding inductively as new concepts emerged. Three authors piloted and finalized a codebook (Figure 1), and then author pairs independently extracted and adjudicated data to ensure reliability.

**Figure 3:**
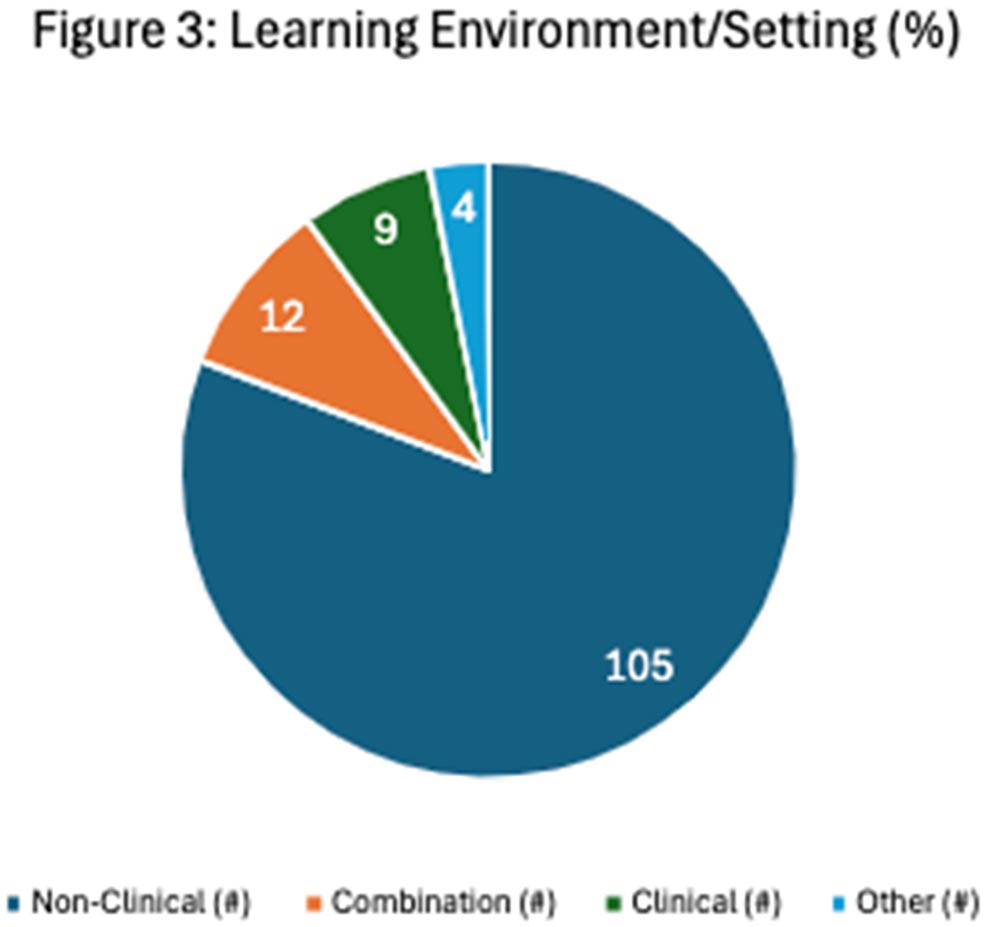
Learning Environment Setting

Figure 3 describes the distribution of abstracts based on the learning environment in which they were implemented. Clinical environment includes clinical rotations and patient care settings, non clinical environment includes teaching and learning spaces outside of patient care settings.

**Figure 4:**
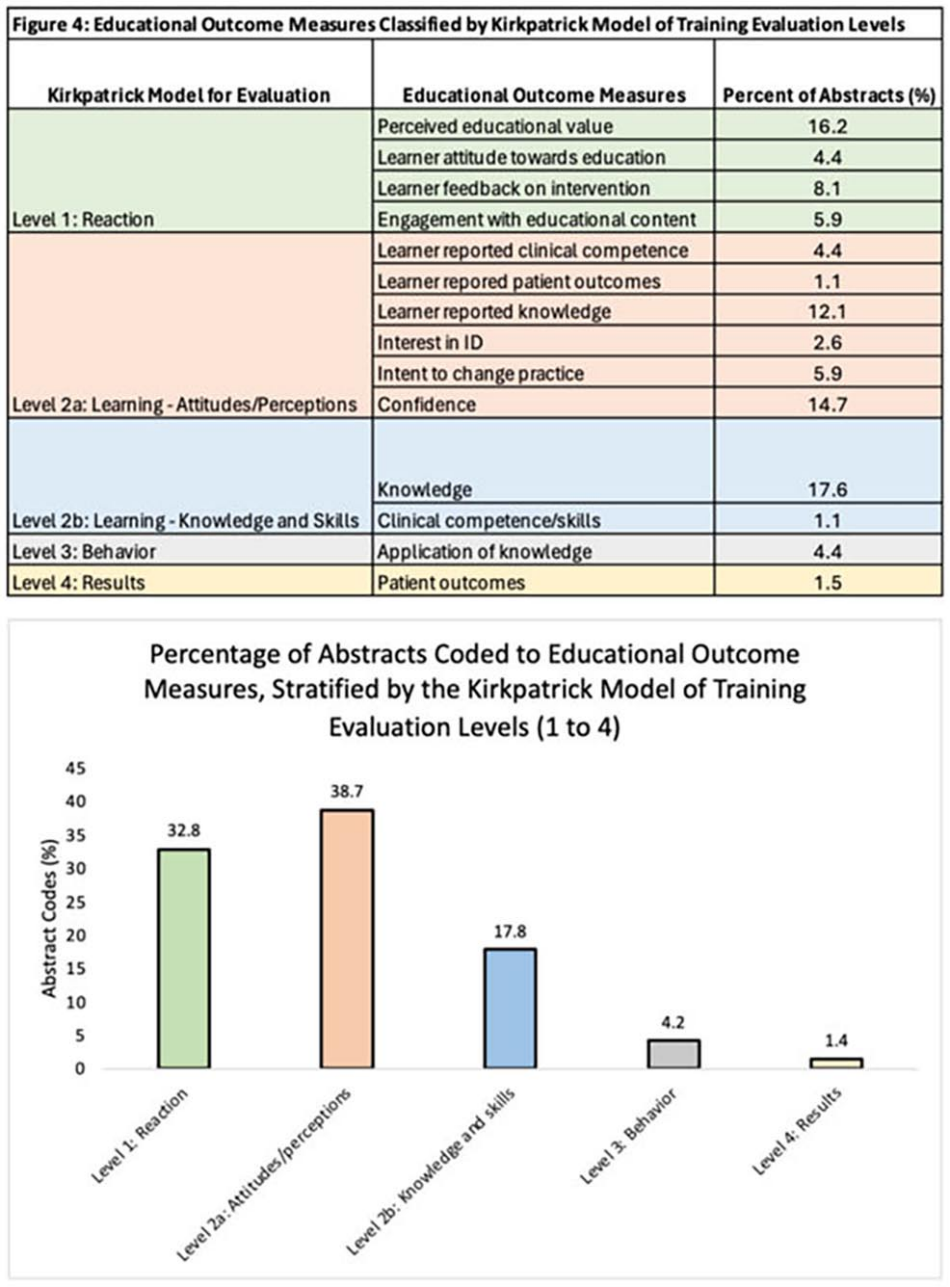
Educational outcome measures classified according to the Kirkpatrick's model for training evaluation levels.

Figure 4 describes educational outcome measures classified according to the Kirkpatrick's model of training evaluation levels.

**Results:**

Of 182 MedEd abstracts published in Open Forum Infectious Diseases, 130 (71.4%) assessed MedEd innovations and were included. Key topics focused on a breadth of content areas (Figure 2). Students and residents were the most common target learners (74 abstracts); fewer studies involved ID fellows (26 abstracts). A broad range of instructional strategies and technologies were employed (Figure 2). Over half the studies were single-site (58%), with the majority of innovations (81%) being implemented in non-patient care environments (Figure 3). Outcomes were mapped across Kirkpatrick’s levels of evaluation (Figure 4).

**Conclusion:**

This review demonstrates the breadth and depth of ongoing ID MedEd innovations, identifies gaps (e.g., transplant ID, mycology, patient education, robust study designs, rigorous statistical analysis, Kirkpatrick evaluation levels 3–4), and reveals the underrepresentation of fellows as learners. These findings highlight opportunities for future research and the development of a multi-institutional ID MedEd research consortium to select research priorities and elevate the impact of MedEd research for our field. This data repository of current and past MedEd Innovations can help support collaboration, promote dissemination, reduce redundancy, crowdsource MedEd resources (e.g., existing online modules, Artificial Intelligence programming) and provide opportunity for networking and community building within ID. This information can be used as a foundation by professional ID societies to allocate future training resources for medical educators and MedEd scholarship.

**Disclosures:**

All Authors: No reported disclosures

